# The Emerging Role of RNA Modifications in the Regulation of Antiviral Innate Immunity

**DOI:** 10.3389/fmicb.2022.845625

**Published:** 2022-02-03

**Authors:** Jie Tong, Wuchao Zhang, Yuran Chen, Qiaoling Yuan, Ning-Ning Qin, Guosheng Qu

**Affiliations:** ^1^College of Life Sciences, Hebei University, Baoding, China; ^2^Institute of Life Sciences and Green Development, Hebei University, Baoding, China; ^3^College of Veterinary Medicine, Hebei Agricultural University, Baoding, China

**Keywords:** RNA modification, viral infection, RIG-I, IFN-I, antiviral innate immunity

## Abstract

Posttranscriptional modifications have been implicated in regulation of nearly all biological aspects of cellular RNAs, from stability, translation, splicing, nuclear export to localization. Chemical modifications also have been revealed for virus derived RNAs several decades before, along with the potential of their regulatory roles in virus infection. Due to the dynamic changes of RNA modifications during virus infection, illustrating the mechanisms of RNA epigenetic regulations remains a challenge. Nevertheless, many studies have indicated that these RNA epigenetic marks may directly regulate virus infection through antiviral innate immune responses. The present review summarizes the impacts of important epigenetic marks on viral RNAs, including N6-methyladenosine (m^6^A), 5-methylcytidine (m^5^C), 2ʹ-O-methylation (2ʹ-O-Methyl), and a few uncanonical nucleotides (A-to-I editing, pseudouridine), on antiviral innate immunity and relevant signaling pathways, while highlighting the significance of antiviral innate immune responses during virus infection.

## Introduction

Chemical modifications of RNA, also be designated as epitranscriptomic marks of RNA, are considered common features in most natural RNAs. To date, more than 140 posttranscriptional modifications have been discovered to function in the structural diversity and metabolism of RNAs (Zhao et al., 2017). While chemical modifications mainly appear in cellular RNAs such as messenger RNA (mRNA), ribosomal RNA (rRNA), and transfer RNA (tRNA) as well as other non-coding RNAs, numerous studies have indicated the pivotal roles of RNA epigenetic regulations in virus infection (McIntyre et al., 2018; Netzband and Pager, 2020). The most prevalent modifications in the virus genome include methylation of adenine and cytidine residues, such as N6-methyladenosine (m^6^A), 5-methylcytidine (m^5^C), or 7-methylguanosine (m^7^G), 2ʹ-O-methylation (2ʹ-O-Methyl), as well as uncanonical nucleotides like A-to-I editing and pseudouridine (McIntyre et al., 2018). Although these chemical modifications are generally formed by cellular enzymes, virus-encoded methyltransferases have been implicated in several methylation modifications. Nearly all chemical modifications that are mediated by enzymes undergo dynamic and reversible changes during virus infection, which makes it difficult to define roles of epigenetic modifications in viral RNA metabolism or virus infection. Nevertheless, due to the rapid development of RNA biology, numbers of RNA modifications have been found in genome of various viruses, which are supposed to influence virus infection to some extent (Courtney, 2021; Marchand and Motorin, 2021).

As the primary antiviral strategies, innate immune responses are invariably activated at the early stage of virus infection. Through recognizing the exogenous nucleic acids including virus-derived RNAs or DNAs by Toll-like receptors (TLRs; Creagh and O’Neill, 2006; Beutler, 2009; Lavelle et al., 2010), which belong to pattern-recognition receptors (PRRs), cytoplasmic receptors/adapters like myeloid differentiation factor-88 (MyD-88) or TIR-domain-containing adaptor protein inducing interferon-beta (TRIF) is recruited and in turn activates TNF receptor-associated factors (TRAFs; Creagh and O’Neill, 2006). Activation of TRAFs then gives rise to the activation of IFN response factor 3/7 (IRF3/7) and nuclear factor-κB (NF-κB) signaling pathways that induces type I interferons (IFNs) and proinflammatory cytokines expression (Bonizzi and Karin, 2004; Rius et al., 2008; Dev et al., 2011). Aside from the TLR pathway, another kind of PRRs named as retinoic acid-inducible gene I (RIG-I)-like receptor (RLR) family also has been identified as crucial cytosolic sensors of viral nucleic acids (Schustak et al., 2021). The mitochondrial antiviral-signaling protein (MAVS) is located in mitochondria or endoplasmic reticulum (ER) and considered as the receptor protein of RLR signaling pathway, by which IFN-β is effectively expressed at the early stage of virus infection (Hwang et al., 2013; Tong et al., 2021b). Both IFNs and proinflammatory cytokines have strong antiviral activities. A battery of studies have recently indicated the emerging roles of RNA modifications in regulating antiviral innate immune responses (Thompson et al., 2021). The present review will focus on the impacts of these epigenetic marks, especially on antiviral innate immunity and its relevant signaling pathways, while highlighting the significance of antiviral innate immune responses during virus infection.

## Prevalent RNA Modifications in Virus

### N6-Methyladenosine

N6-Methyladenosine modification affects nearly all aspects of RNA biology, including stability, translation, splicing, nuclear export, and localization. Methylation modification, adding adenosine to N6 to form m^6^A, is catalyzed by a large heterogeneous complex of proteins that are named as “writer,” including METTL3, METTL14, or Wilms tumor 1-associated protein and KIAA1429 (Meyer and Jaffrey, 2017; Shi et al., 2019). In contrast, demethylases enzymes like fat mass and obesity-associated protein (FTO) or α-ketoglutarate-dependent dioxygenase AlkB homology 5 (ALKBH5) designated as “eraser” remove the methyl group (Jia et al., 2011; Zheng et al., 2013). The YTH domain family of proteins (YTHDC1, YTHDC2, YTHDF1, YTHDF3, and YTHDF3) and others named as “reader” recognize and bind to the m^6^A modification site to directly regulate the posttranscriptional functions of modified RNAs (Shi et al., 2019). m^6^A modifications are typically identified within the DRA^m^CH motif (D = G/A/U, R = A/G, and H = A/C/U); however, given the fact that only some of the DRACH motifs in eukaryote transcriptome are modified, there might exist some mechanisms for site-selective modification (Dominissini et al., 2012).

### 5-Methylcytidine

Another kind of RNA base methylation is the C5-methylation of RNA cytosine-m^5^C. m^5^C widely exists in cytoplasmic and ribosomal RNA (rRNA), tRNA, mRNA, and some non-coding RNAs (Lewis et al., 2017; Bohnsack et al., 2019). In eukaryotes, m^5^C is catalyzed by enzymes of the NOL1/NOP2/SUN domain (NSUN) family and DNA methyltransferase family protein (DNMT2), a homolog of DNA methyltransferase (Reid et al., 1999). Recent studies showed m^5^C was present in numerous virus genomes and might have non-negligible effects on antiviral innate immunity (Winans and Beemon, 2019; Wnuk et al., 2020). For instance, a high level of m^5^C modifications in HIV-1 genomic RNA (gRNA) promoted the expression of viral genes by regulating splicing and the translation efficiency of viral mRNAs (Courtney et al., 2019). Silencing or inactivation of the major writer NSUN2 of m^5^C reduced the m^5^C abundance in HIV-1 transcripts and inhibited virus replication by disrupting the alternative splicing and the followed translation of HIV-1 mRNA (Kong et al., 2020).

### 2ʹ-O-Methylation and 7-Methylguanosine

Cellular mRNA conventionally has a triphosphate at 5ʹ end (5ʹ-ppp), which is converted to 5ʹ-diphosphate (5ʹ-pp) by RNA triphosphatase (Ramanathan et al., 2016). This conversion resulted in mRNA capping by guanylyltransferase and guanine-N7 methyltransferase (Shatkin, 1976). After adding a terminal guanosine base, the mRNA transcripts possess an m^7^G joined *via* a 5ʹ, 5ʹ-triphosphate bridge, designated as cap-0 (Shatkin, 1976). When a cellular 2ʹ-O-methyltransferase, CMTR1, further modifies the mRNA, a methyl group is added at the 2ʹ-O-hydroxyl position of the first nucleotide to form cap-1 RNA structure (Belanger et al., 2010). Meanwhile, a second 2′-O-methyl group can be added at the second nucleotide to form cap-2, catalyzed by another cellular methyltransferase CMTR2 (Werner et al., 2011; Smietanski et al., 2014). mRNA capping is considered one of the key factors in regulating RNA metabolism and function (Topisirovic et al., 2011), including stabilizing the mRNA and serving as a chemical marker to discriminate self from foreign RNA, the latter of which may interfere with the innate immune sensing of viral derived RNA (Hocine et al., 2010; Ramanathan et al., 2016). Some viruses, such as West Neil virus (WNV) or Dengue virus (DENV), encode 2ʹ-O MTases that catalyze 2ʹ-O-methyl adenosines inside the virus genome (Dong et al., 2012; Chang et al., 2016). Interestingly, this internal adonosine 2ʹ-O-methyl activity requires the same K-D-K-E motif as that for 2ʹ-O methylation of the 5ʹcap (Yap et al., 2010; Dong et al., 2012). Given that many viruses possess cap structures in their RNA components, this type of modification is supposed to play a pivotal role in antiviral innate immunity.

### Uncanonical Nucleotides

After the pseudouridine (ψ) was firstly identified in plant turnip yellow mosaic virus (TYMV) in 1998 (Becker et al., 1998), the follow-up research continuously indicated the abundant ψ in RNA viruses, especially in positive-sense RNA viruses (McIntyre et al., 2018). Psedouridine occurs through isomerization of uridine-to-5-ribosyl uracil by pseudouridine synthases (PUS; Markham and Smith, 1951). Similar to ψ, the deamination of adenosine to inosine (A-to-I) that depends on the catalyzing of adenosine deaminase acting on RNA (ADAR) family is also considered RNA editing or uncanonical nucleotides (Chen et al., 2000; Bass, 2002; George et al., 2014; Chung et al., 2018; Eisenberg and Levanon, 2018). The difference is that inosine generally acts similarly to guanosine (G), whereas ψ remains the original capacity of uridine to some extent. Although U to ψ conversion does not change the Watson–Crick base-pairing with adenosine, in certain cases, ψ enables base pairing with any other nucleotides (Samuel, 2011; Pfaller et al., 2021). Both ψ and A-to-I editing may significantly convert RNA biology, including changing the coding preference of viral RNA dependent RNA polymerases, mediating alternative splice and even affecting RNA structures (Netzband and Pager, 2020; Pfaller et al., 2021).

### Mechanisms of Epigenetic Regulation in RNA Metabolism

**Figure 1:**
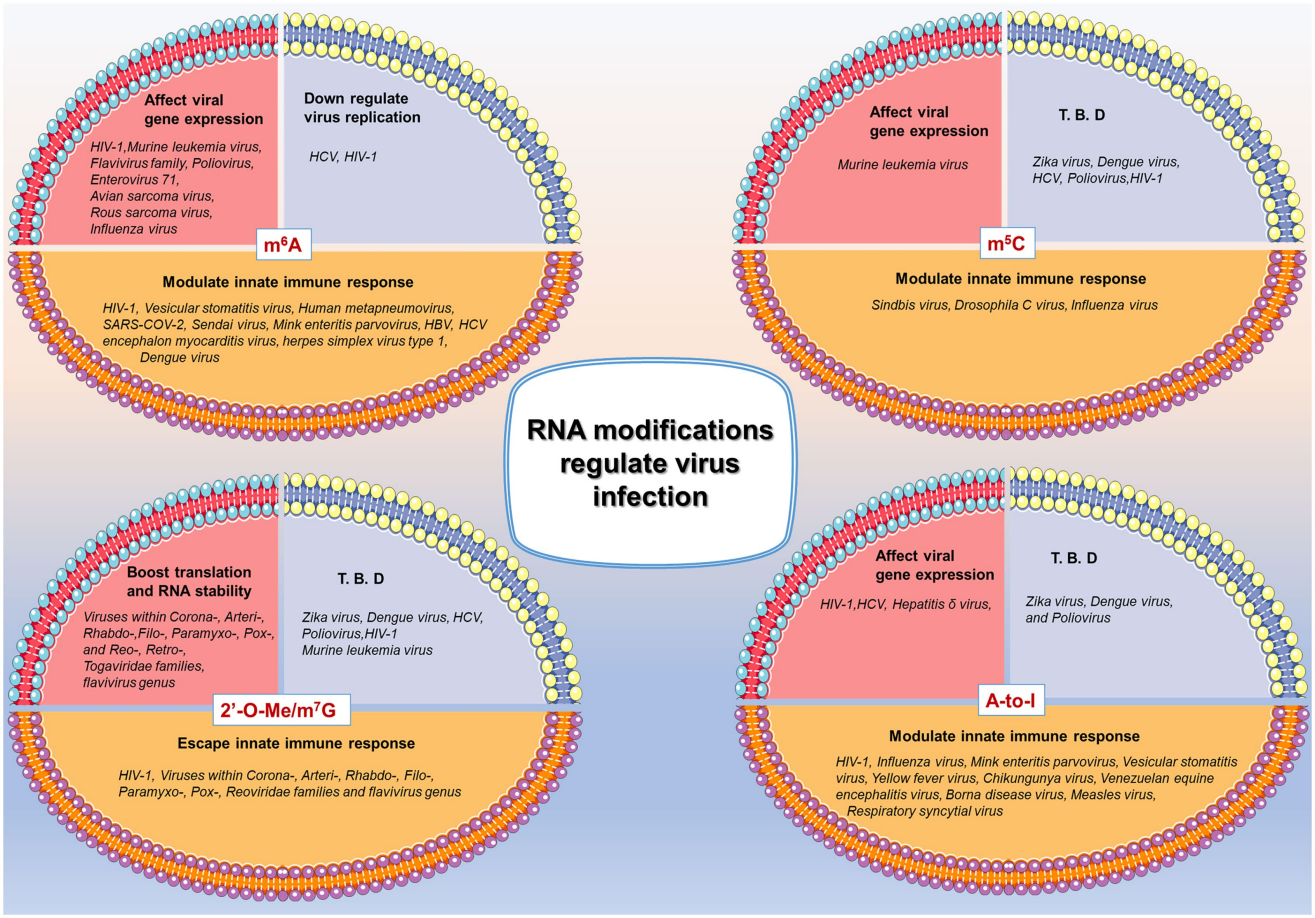
Brief summary of RNA modifications in regulating virus infection. N6-methyladenosine (m^6^A), 5-methylcytidine (m^5^C), 2ʹ-O-methylation (2ʹ-O-Me)/7-methylguanosine (m^7^G), and A-to-I editing (A-to-I) are listed as example of epigenetic regulations in virus infection. Three major effects are summarized in diverse virus infection, among which the modulation of innate immune response is the focus of the present review. T. B. D: To be determined. References are listed in the Supplementary Table S1.

Modified nucleotides may stabilize the functional RNA structures by reinforcing the hydrogen bond between Watson–Crick pairs, resulting in augmented thermal stability and reduced dynamics (Serra et al., 2004; Zhou et al., 2016; Frye et al., 2018). Under other circumstances, Watson–Crick base pairs consisting of modified nucleotides may induce an alternative folding representing significant alterations in the RNA secondary or tertiary structures, negatively affecting RNA stability (Durbin et al., 2016; Zhou et al., 2016; Roundtree et al., 2017; Wei and He, 2021). Moreover, the posttranscriptional introduction of modified nucleotides can affect RNA intermolecular interactions with other encountered molecules such as DNA partners, RNA binding proteins, or other RNAs (Zhao et al., 2017; Nachtergaele and He, 2018; Shi et al., 2019). Since all functions are regulated by structure to a certain degree, viral RNAs carrying modified nucleotides (or uncanonical nucleotides) commonly represent functional differences in virus life-cycle, thus mediating virus infection in host cells. Besides, some RNA viruses those complete their life-cycle in cytoplasm influence host cell genomic transcription inside the cell nucleus, for example, ZIKV infection affects some endogenous genes’ trancription which occured inside the cell nucleus (Gokhale et al., 2020). In this case, epigenetic regulations may facilitate the virus to overcome the spatial barrier. To sum up, some of the demonstrated RNA modifications in virus genome as well as the correlative functions are listed in Figure 1. Notably, many viruses use epigenetic modifications as crucial tools to evade antiviral innate immune response.

## RNA Modifications in Sensing of Foreign Nucleic Acids

**Figure 2:**
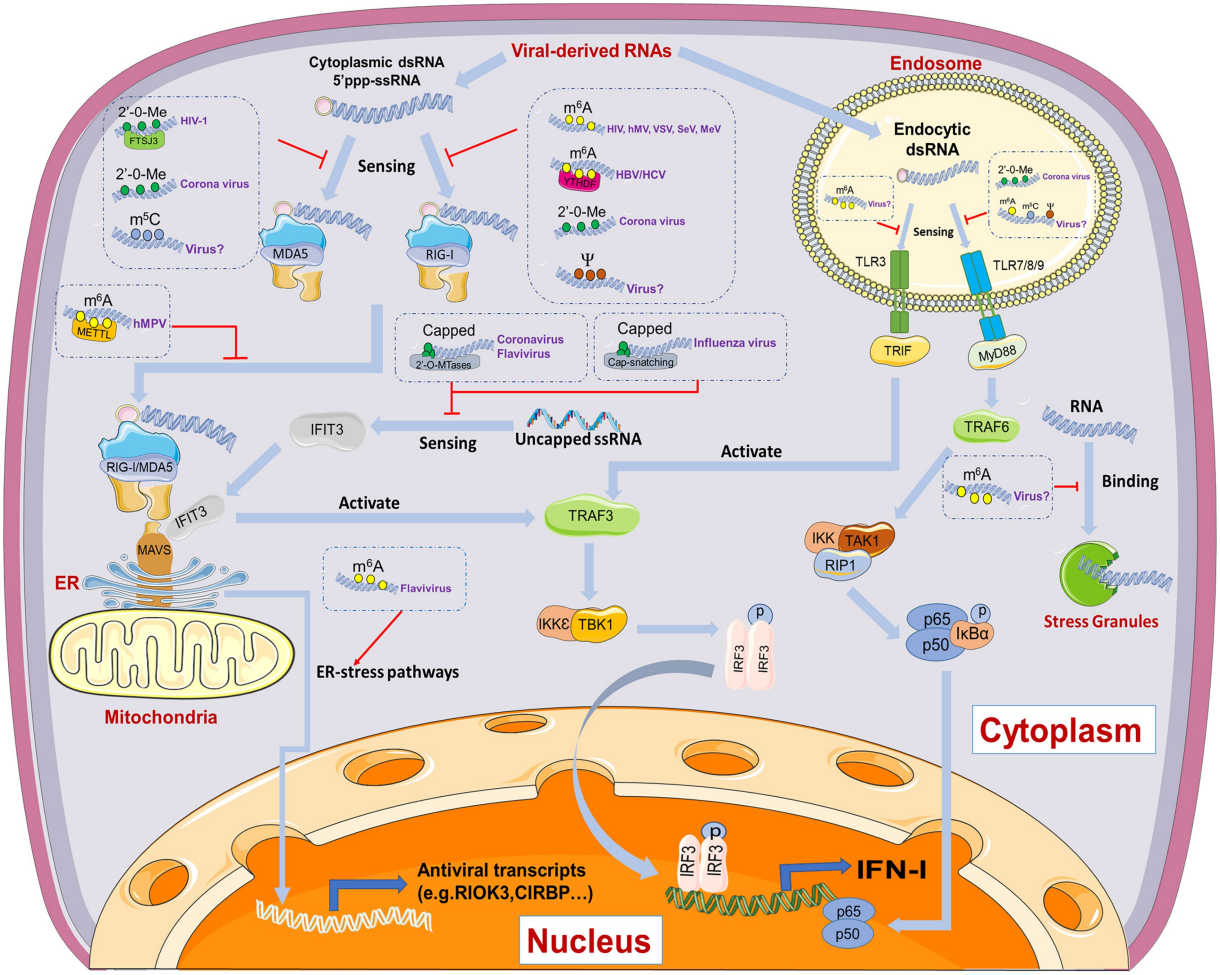
Schematic diagram of mechanisms by which RNA modification regulating viral-derived RNA recognition and innate immune responses. N^6^-methyladenosine (m^6^A), 5-methylcytidine (m^5^C), 2ʹ-O-methylation (2ʹ-O-Me), and pseudouridine (ψ) are demonstrated to inhibit melanoma differentiation-associated protein 5 (MDA5) or retinoic acid-inducible gene I (RIG-I) mediated sensing. METTL and YTHDF proteins are involved in these process. Inside the endosomes, 2′-O-Me are identified to block the TLR7-dependent type I interferon (IFN-I) response. Meanwhile, m^6^A, m^5^C, and ψ also prevent the Toll-like receptors (TLRs) activation inside the endosomes, although the relevance to virus infection still remains ambiguous. Moreover, m^6^A may regulate antiviral innate immunity through stress granules or endoplasmic reticulum (ER)-stress pathways, the latter of which has already been illustrated in Flavivirus infection.

Sensing the foreign molecules by the PRRs of the innate immune system serves as the initial step of the innate immune response (Akira et al., 2006; Takeuchi and Akira, 2010). Different PRRs must distinguish the non-self molecules from the self through chemical patterns. To date, several kinds of PRRs, including TLRs, RLRs, cyclic GMP-AMP synthase (cGAS; Schoggins et al., 2014; Aguirre et al., 2017; Ma et al., 2021; Yu et al., 2021), C-type lectin receptors (CLRs), nucleotide-binding oligomerization domain (NOD)-like receptors (NLRs), and AIM2-like receptors (ALRs), have been utilized by host cells in recognition of viral PAMPs (Akira et al., 2006; Crowl et al., 2017; Babamale and Chen, 2021; Chou et al., 2021; de Oliveira Mann and Hornung, 2021). As the most important component in virus particles, viral-derived RNAs/DNAs are released into the host cell cytoplasm at the early stage of infection. Thus, recognizing the distinction of epitranscriptomic modifications between cellular and pathogen nucleic acids is supposed to regulate antiviral innate immunity at the early stage of virus infection (Figure 2).

### Retinoic Acid-Inducible Gene I-Like Receptors

Retinoic acid-inducible gene I, melanoma differentiation-associated protein 5 (MDA5), and laboratory of genetics and physiology 2 (LGP2) are the three major homologous helicases of RLRs (Wu et al., 2013; Yu et al., 2018; Thoresen et al., 2021). RIG-I and MDA5 displayed similar component structures, including caspase activation domain in N-terminal, recruitment domains (CARDs) for communicating with downstream signals, a DExD/H-box helicase domain with RNA binding and ATP hydrolysis activity, and a C-terminal domain (CTD; Yoneyama et al., 2004; Jiang et al., 2011; Kowalinski et al., 2011; Yu et al., 2018). The pathogen-associated molecular patterns (PAMPs) motifs of RIG-I include exposed 5ʹtriphosphate (5ʹppp) or diphosphate of double-strand RNAs (dsRNA), panhandle structures of viral genomic RNA, and uridine-rich sequences, while MDA5 recognizes long dsRNA such as poly (I:C; Yoneyama et al., 2004; Hornung et al., 2006; Kato et al., 2006; Cui et al., 2008; Liu et al., 2018). RIG-I is expressed at a low level in non-infected cells, usually referred to as a resting state with RNA-binding and helicase domains covered by RDs (Kowalinski et al., 2011; Luo et al., 2011). Following recognition of PAMPs, RIG-I undergoes a conformational change that provides room for closer interaction with more PAMPs RNAs and begins to release the CARDs for MAVS interaction and signaling (Pichlmair et al., 2006; Peisley et al., 2013; Thoresen et al., 2021). When the complex consisting of RIG-I, MAVS, and other cytosolic proteins translocate from the cytoplasm to the associated mitochondrial membrane, RIG-I CARDs interact with the MAVS CARD to catalyze the filament formation of MAVS and then activates TBK1 and IKKε to initiate downstream signaling (Pichlmair et al., 2006; Peisley et al., 2013; Goubau et al., 2014).

#### m^6^A and RLRs

As one of the most common RNA modifications, m^6^A has been widely involved in the innate immune sensing process and thus regulates viral pathogenesis. In some cases, viral RNA-loaded m^6^A modification dampens the RIG-I mediated RNA sensing and activation of the downstream transcription factors such as IRF3 and IRF7, which depresses the type I interferon (IFN-I) gene expression (Lu et al., 2020; Ge et al., 2021; Xue et al., 2021). One suggested mechanism is that m^6^A modification might harbor viral PAMPs motifs for RIG-I recognition and innate immune signaling. For example, m^6^A modifications in HIV RNAs undermined RIG-I sensing and type-I interferon induction in differentiated monocytic cells, while m^6^A-deficient HIV-1 virions produced from FTO-overexpressing HEK293T cells induced high levels of IFN-I expression in a RIG-I-dependent manner (Chen et al., 2021). Consistently, in several families of negative-sense RNA viruses, such as Pneumoviridae (hMPV), Paramyxoviridae (SeV and MeV), and Rhabdoviridae (VSV), m^6^A-deficient viral RNAs universally triggered RIG-I-dependent innate immune response much more efficiently compared to the m^6^A-sufficient viral RNAs, suggesting a crucial role of m^6^A marker in RIG-I sensing process (Kim et al., 2020; Lu et al., 2020, 2021).

Further investigation demonstrated that these negative effects might be induced by m^6^A related enzymes, including YTHDFs and METTLs. Instead of encoding innate immune antagonist proteins, m^6^A modifications in viral RNAs enable the recruitment of the m^6^A enzymes, which subsequently sequestrates viral ds/ssRNA through their RNA binding ability to prevent RIG-I recognition. Specifically, m^6^A modification of hepatitis B and C viral RNAs suppressed the activation of RIG-I signaling, whereas single nucleotide mutation of m^6^A motif of viral RNAs (A8766C) enhanced RIG-I sensing activity (Kim et al., 2020). In this case, YTHDF2 was found to regulate HBV pgRNAs and HCV genomic RNAs to evade RIG-I recognition. Besides YTHDF itself, diverse RNA-binding proteins (RBPs) were identified to interact with YTHDF proteins. The interactions might also regulate RIG-I access to viral RNAs, which mediates the activation of RIG-I signaling pathways through indirect influences (Luo et al., 2012).

Moreover, reducing the m^6^A “writer” enzyme METTL3 not only downregulates the m^6^A levels in the 3ʹ end of SARS-COV-2 genome, but also improves the RIG-I binding to enhance the downstream innate immune signaling pathway and inflammatory gene expressions (Li et al., 2021). Similarly, as for vesicular stomatitis virus (VSV) infection, METTL3 decreases viral dsRNA formation, thereby impeding virus-sensing efficacy by RIG-I and dampening antiviral immune signaling (Qiu et al., 2021). However, due to the lack of precise information about RIG-I PAMPs, the universal mechanisms of viral RNA m^6^A modification inhibiting RIG-I activation remains unclear. A potential clue has been elucidated in human metapneumovirus (hMPV) infection. Due to the indispensable role of conformational change in RIG-I activation, m^6^A modifications in virus genome might block the binding of viral RNAs to RIG-I, which disabled the conformational change of RIG-I, as well as the subsequent MAVS-TBK1 pathways. In this case, m^6^A-deficient hMPV virion RNA induced much higher RIG-I expression (Lu et al., 2020).

#### 2ʹ-O-Methylation and RLRs

Besides m^6^A modifications, RNA 2ʹ-O-methyl is a highly conserved process used by RNA viruses to evade sensing by cytosolic RNA sensor proteins (Daffis et al., 2010; Decroly et al., 2011; McFadden et al., 2017). Early studies indicated that the 2ʹ-O-methyl commonly marks viral RNA as “self,” which prevents RLRs and downstream signaling pathways (Hyde and Diamond, 2015; McFadden et al., 2017; Jaafar and Kieft, 2019). During HIV-1 infection, viral RNAs were methylated to carry internal 2ʹ-O-methylations by the cellular methyltransferase FTSJ3 (Ringeard et al., 2019). When HIV-1 viruses were produced in FRSJ3 knock-out cells, the induction of IFNs was heavily enhanced in an MDA5-dependent manner (Zust et al., 2011; Ringeard et al., 2019). Similarly, 2ʹ-O-methyls on the coronavirus family viral RNAs also perturbed type I interferon production that is dependent on either the MDA5 or RIG-I sensing process (Zust et al., 2011; Devarkar et al., 2016).

Additionally, the virus facilitated the capping of viral RNAs at the 5ʹ terminal to disturb the innate immune sensing process (Bradrick, 2017; De Vlugt et al., 2018). Unlike cellular mRNA transcripts, some viruses, including flaviviruses and coronaviruses, encode enzymes with m^7^G and 2ʹ-O-methyltransferase (2ʹ-O-MTases) activity to cap their RNA, It has been showed that 2ʹ-O-MTases-deficient virus are highly sensitive to IFN-I (Chen and Guo, 2016; Bradrick, 2017). Although, the precise factors that sense unmethylated RNAs as invading nucleic acid are still unclear, the interferon-induced protein with tetratricopeptide repeats (IFIT) family has been discovered to function in West neil virus, poxvirus, and coronavirus infection (Daffis et al., 2010). Interestingly, instead of encoding 2-O-MTases, the influenza virus applies a “cap-snatching” strategy to ensure the viral RNA 5ʹ end modifications that prevent the viral RNA from being sensed by IFIT proteins (De Vlugt et al., 2018).

#### Other RNA Modifications and RLRs

Along with m^6^A modifications and 2ʹ-O-methyl, other RNA chemical modifications also participate in RLRs sensing-dependent innate immune response (Ahmad et al., 2018). For example, RIG-I and MDA5 detection of dsRNA is blocked by adenosine deaminase acting on RNA (ADAR1), which catalyzes RNA A-to-I modification (Mannion et al., 2014; Yang et al., 2014; Ahmad et al., 2018; Tang et al., 2021). Although it is well-demonstrated how ADAR1-mediated A-to-I modifications impeded MDA5 activation in the mouse study (Liddicoat et al., 2015; de Reuver et al., 2021), MDA5-dependent sensing has rarely been found in A-to-I editing-induced innate immune response. In other cases of A-to-I editing in virus infection, suppression of innate immune IFN responses after virus infection is mainly mediated by cytoplasmic dsRNA sensors protein kinase R (PKR) and oligoadenylate synthetase (OAS; Yang et al., 2014; Radetskyy et al., 2018; Lamers et al., 2019). Rather than upstream dsRNA sensors, PKR, and OAS are identified as pivotal antiviral IFN stimulated genes (ISG). Thus, more details about ADAR1-mediated A-to-I modifications in antiviral innate immune response will be further discussed below.

Similar to A-to-I editing, pseudouridine modifications were shown to abolish RIG-I’s filament formation and PAMPs RNA’s binding (Peisley et al., 2013). Given the abundant pseudouridine modifications in RNA viruses, especially the positive-sense RNA viruses (McIntyre et al., 2018), this type of uncanonical nucleotides is suggested to regulate various aspects of the antiviral response.

### Toll-Like Receptors and Other PRRs

Another well-characterized PRRs, TLRs, are widely distributed invertebrates. TLRs are anchored in the cell membrane as type I transmembrane proteins (Akira et al., 2006). The ectodomain (N-terminal) of TLRs consists of several leucine-rich repeat (LRR), which connect to the C-terminal Toll/interleukin-1 receptor (TIR) domain by transmembrane (TM) domain (Akira et al., 2001; Kawasaki and Kawai, 2014). Studies have shown that most TLRs function as homology dimers (Kawai and Akira, 2011). Two TIR domains became close to forming a competent signaling state that recruits the adapter proteins (O’Neill et al., 2013). Nearly all the activated TLRs can trigger proinflammatory gene expression despite functioning in specific aspects of antiviral immunity (Kawai and Akira, 2011). To date, 10 TLRs have been identified in human cells. Four of them functioned as immune sensors by detecting pathogens-derived nucleotides (Kawasaki and Kawai, 2014). TLR3 recognizes long dsRNA and recruits TRIF as its dedicated adapter protein. Phosphorylated TRIF provides a signaling hub for IRF3 phosphorylation by TBK1, which then activates downstream signaling pathways of TRIF (Matsumoto et al., 2011; Oshiumi et al., 2011; Liu et al., 2015). TLR7 and TLR8 detect RNA debris as short RNA segments, while TLR9 enables sensing short DNA fragments that contain CG dinucleotide motifs (Chan et al., 2015; de Oliveira Mann and Hornung, 2021). TLR7, 8, and 9 can recruit the adapter protein MyD88 to form a complex known as the Myddosome. Myddsosome interact with IκB kinase and TGF-beta-activated kinase 1 (TAK1) complex to initiate NF-κB and MAPK signaling, respectively (Motshwene et al., 2009). Interestingly, the complex can also trigger IRF activation that depends on TASL that is only expressed in specific cells (Heinz et al., 2020). TASL is also capable of IRF phosphorylation, while in this case, IRF5 and IRF7, as well as IRF3, may be activated to drive antiviral gene expression (Wust et al., 2021).

Although several studies indicate the important roles of TLRs that usually sense long dsRNA inside endolysosome or outside the cells in antiviral innate immune response, they have rarely been found to be regulated by RNA modifications, partly because many RNA viruses expose their genomic dsRNA in the cytoplasm (Akira et al., 2001; Alexopoulou et al., 2001; Heil et al., 2004). Some studies have implied that the epigenetic marks of viral RNA interfere with the innate immune signaling pathway by preventing TLRs activation. For instance, the 2ʹ-O-methyl marks on coronavirus RNAs avoid the recognition of TLR7 to evade the activation of the IFN signaling pathway, while this effect may also be achieved through MDA5 sensing signals (Zust et al., 2011). Coronaviruses replicating in MDA5 or TLR7 deficient mice are detected to the same extent as in IFNR-deficient mice. By employing *in vitro* modified RNA oligos, an early study showed that m^6^A limited the capacity of RNAs to activate TLR3, TLR7, and TLR8, while m^5^C and Ψ blocked the activation of TLR7 and TLR8 (Kariko et al., 2005). Recent studies applied CRISPER tools to map the function of m^6^A and demonstrated that m^6^A could suppress macrophage activation through TLR mediated signaling (Tong et al., 2021a). However, more evidence of virus RNA modifications regulating TLR mediated pathways in innate immune response remains to be discovered.

## RNA Modifications in Regulating IFN Signaling Pathway

Interferon is a group of signal proteins synthesized and released by host cells in response to stress and infections. Interferon exists widely in human and other animal organisms with highly species specifictiy (Crow and Stetson, 2021). According to the types of corresponding receptors, interferon can be divided into three types: IFN-I, type II interferon (IFN-II), and type III interferon (IFN-III; Hervas-Stubbs et al., 2011; Stanifer et al., 2019). After infected with viruses, cells release IFNs to restrict the virus infection and even degrade the virions. Although IFNs do not kill the virus directly, IFNs enable the transcription and production of several enzymes that interfere with the viral genome transcription or translation of viral protein components (Sadler and Williams, 2008).

Meanwhile, IFNs also improve the antiviral ability of the surrounding cells. Therefore, IFNs are commonly considered powerful tools and key components in the first line of innate immune defense against viruses infection.

Interferons function mainly through the interactions between IFN molecules and cell surface receptors. Upon specifical recognition and binding by IFNs, the IFN receptors undergo conformational changes, activating the JAK family proteins and promoting the recruitment and phosphorylation of signal transduction and transcriptional activation (STAT) proteins. The phosphorylated STAT is then dimerized and binds to IRF9 to form an ISGF3 complex, a transcriptional factor after transfer into the nucleus. The ISGF3 regulates the expression of numerous kinds of IFN stimulating genes ISGs, which exert strong antiviral effects (Darnell, 2012; Raftery and Stevenson, 2017). However, many viruses (e.g., SARA-COV-2 or influenza virus) encode structural and non-structural viral proteins that ablate the IFN signaling pathways through interaction with other cellular signaling pathways. This usually results in invalid STAT that fails to form phosphorylated ISGF3 complex, further abolishing the expression of antiviral ISGs (Mazewski et al., 2020; Yin et al., 2020; Jung and Lee, 2021). This process is concluded as an evasion of the innate immune response. Evading of the IFN-dependent innate immune response also relates to persistent infections. For example, direct binding of the Borna disease virus (BoDV) encoded P protein to TBK1 can antagonize the IRF3 activation, which prevents IFNβ induction (Unterstab et al., 2005). It is hypothesized that the ability of BoDV to prevent IRF3-dependent genes transcription might prevent the virus from activating the RLR signaling pathway and give rise to persistent BoDV infections in mammalian and avian hosts (Peng et al., 2007).

Whenever ISGs are successfully expressed, they will perform diverse antiviral effects. More than an important effector in IFN-dependent antiviral immune response, some ISGs can also be upregulated directly and independent of IFNs after virus infection. Although ISGs have different effects, on the whole, they all can resist or control infectious pathogens (Schneider et al., 2014; Fensterl et al., 2015). Previous studies showed that ISGs generally functioned by interacting with different co-factors, mediating antiviral effects by promoting viral RNA degradation, abrogating viral proteins translation, or combining both (Nguyen et al., 2001; Bick et al., 2003; Yang and Li, 2020). Moreover, secreted IFNs and induced ISGs may also activate NF-κB or other related innate immune signaling pathways to improve the release of proinflammatory cytokines and/or induce apoptosis that further restricts virus infection (Peteranderl and Herold, 2017).

### m^6^A in IFN Producing and Effecting

The biological function of m^6^A is mainly regulated by a methyltransferase (writer), demethylase (eraser), and m^6^A binding protein (reader; Tong et al., 2018). Many studies have shown that RNA m^6^A modification plays an important role in innate immune response, while the exact roles of m^6^A in regulating antiviral IFN signaling displays in opposite aspect (Gokhale et al., 2016; Guo et al., 2020). In some cases, m^6^A modifications in the virus genome promote the IFN and ISGs induction, whereas, under other circumstances, m^6^A modification occurs to turn off the antiviral innate immune response. For example, the m^6^A modifications at specific sites in the HBV transcript restricts the virus replication through IFN α-mediated response. Although HBV is a DNA virus, it replicates through transitional pre-genomic RNA (pgRNA). m^6^A modification of A1907 in HBV pgRNA is the key regulator of IFN α-mediated pgRNA decay. Further investigation showed that ISG20 selectively degraded the m^6^A HBV transcripts that are strictly regulated by m^6^A reader YTHDF2 (Liu et al., 2017; Imam et al., 2020).

Contrary effects were found in encephalon myocarditis virus (EMCV), herpes simplex virus type 1 (HSV-1), and VSV infection. In these cases, YTHDF3 can inhibit the expression of ISGs by promoting the translation of transcriptional inhibitor FOXO3 (Zhang et al., 2019). RAW264.7 cells with YTHDF3 gene deletion have extensive antiviral activity against RNA and DNA virus, and this activity is mediated by the IFNAR1 signal (Zhang et al., 2019). Notably, m^6^A modification, in this case, regulated the host cell transcripts to inhibit antiviral innate immune response instead of affecting viral RNAs. Indeed, this viral infection-induced host cell m^6^A epitranscriptome diversity has commonly been found to regulate the antiviral innate immune response. During VSV infection, m^6^A modifications in MAVS, TRAF3, and TRAF6 are demethylated by ALKBH5 through interacting with the RNA helicase DDX46, which leads these three transcripts to retention in nuclei. Abolished expression of these three transcripts prevents efficient IFN induction (Zheng et al., 2017). Similarly, human cytomegalovirus (hCMV) infection affects host m^6^A modification machinery, including METTL14 and ALKBH5, reducing the IFNβ production. When knocking down the expression of METTL14, the production of IFNβ and subsequent signaling depending on the JAK/STAT pathway are enhanced, which decreases the production of infectious hCMV virion in infected cells (Rubio et al., 2018).

Interestingly, besides the direct influences of m^6^A modification on HBV pgRNA, it also has been indicated that m^6^A modification of tumor suppressor phosphatase and tensin homolog (PTEN) transcript is affected by HBV infection through invaliding PI3K/AKT pathway and inhibiting IRF-3 nuclear export (Kim et al., 2021). Other studies also indicated that DHX58, p65, and IKKγ, which bind to YTHDF2, are mediated by m^6^A modification, potentially interfering with IFN induction during virus infection (Lichinchi et al., 2016). Besides, YTHDF1, METTL3, and METTL14 have also been found to increase the expression of ISGs like IFITM1 in an m^6^A binding-dependent manner, which further indicated the m^6^A methyltransferase complex might promote the antiviral activity of type I IFN (McFadden et al., 2021).

Excluding the direct regulations of host transcripts by m^6^A modification, interactions between RNA and RBPs may also be affected by m^6^A modifications that subsequently affect antiviral IFN response (Bidet et al., 2014). For example, it has been found that during DENV-2 infection, three conserved RBPs, G3BP1, G3BP2, and CAPRIN1, are regulatory factors necessary for antiviral IFN response by promoting the efficient translation of PKR and IFITM2 mRNAs (Arguello et al., 2017; Edupuganti et al., 2017).

### Other RNA Modifications in IFN Producing and Effecting

As one of the most important signaling pathways in innate immune responses, IFN producing and effecting are likely regulated by diversity factors, probably due to numerous protein enzymes evolving in the IFN signaling pathway. For example, NSUN2, the methyltransferase of m^5^C, has multiple effects on RNA biogenesis, including converting vault ncRNA to vtRNA (Bohnsack et al., 2019; Kong et al., 2020). The vtRNA has been shown to promote Influenza A virus (IAV) replication in A549 cells and mouse lungs through repressing PKR activation and the subsequent effects of interferon (Li et al., 2015; Wnuk et al., 2020). Similarly, DNMT2 has been reported to be required for efficient IFN responses in Drosophila C virus or Sindbis virus infected Drosophila (Durdevic et al., 2013; Bhattacharya et al., 2017).

Other studies also indicate an important role of ADARs, the enzymes mediating A-to-I editing, in modulating innate immune response during virus infection (Pfaller et al., 2021). ADAR1 has a proviral effect on Measles virus (MeV) and VSV infection that depends on PKR activation (Nie et al., 2007; Pfaller et al., 2015), while the suppression of innate immune response by ADAR2 is supposed to rely on STAT1 in the case of Chikungunya virus (CHIKV) and Venezuelan equine encephalitis virus (VEEV; Schoggins et al., 2011; Clavarino et al., 2012). In the case of other viruses, such as BoDV, IAV, and Yellow fever virus (YFV), the mechanisms of ADAR-regulated IFN response remain indistinct (Pfaller et al., 2021). Interestingly, during HIV-1 infection, ADAR1 and ADAR2 may have opposite effects on virus replication, through the forming of DNA:RNA heteroduplex or antiviral innate immune response, respectively (Clerzius et al., 2009; Doria et al., 2009; Pujantell et al., 2017).

## RNA Modifications Altered Specific Cellular Transcripts to Regulate Antiviral Responses

Except for the viral RNA modifications, some RNA modification can also directly control the expression of cytokines or specific genes that important for antiviral responses. In Flavivirus infection, the m^6^A abundance of host cell transcripts CIRBP and RIOK3 are altered through ER stress and RIG-I signaling respectively, which further regulate virus infection through antiviral immune response (Gokhale et al., 2016, 2020). m^6^A modification also destroyed the binding of stress granules (SGs) proteins to their RNA partners (Arguello et al., 2017; Fu and Zhuang, 2020). These may explain the diverse function of G3BP1, G3BP2, and CAPRIN1 in virus infection. G3BP1 and CAPRIN1 functioned as proviral factors in vaccinia virus (VACV) and respiratory syncytial virus (RSV) infection, while in contrast, G3BP1 and G3BP2 performed antiviral activity against poliovirus (PV) and alphaviruses (Bidet et al., 2014; Eiermann et al., 2020).

5-Methylcytidine has also been described to affect the expresson of host cell genes, which include cell cycle regulator p21 and immunity-related protein IL-17A (Wnuk et al., 2020). There were also studies suggesting a potential role of m^5^C in regulating other host genes, including those functioning in antiviral response.

## How Epigenetic Marks Regulate Virus Infection?

Compared to the heritable evolutions, epitranscriptomic marks on virus genomes that are controlled by various protein factors including endogenous modifying enzymes undergo more dynamic changes. This type of epigentic regulation has been identified to play important roles in virus-host arms race. On one hand, epigenetic modifications of the virus genome prevent the host from recognizing the viral-derived RNAs, thus invaliding the antiviral innate immune response. On the other hand, the host epitranscriptome profiles may vary with virus infection so as to induce expression of uncanonical antiviral genes that restircts virus replication. Notably, the changes to the host transcriptome likely occur in the late stage of virus infection. As a result, the epigenetic machinery tends to facilitate the virus infection at the early stage. However, the dynamic property of RNA modifications on both virus and host transcriptiomes has even complicated epigenetic regulation of the virus-host arms race. Nevertheless, it is worthwhile to harness epigenetic regulations to intervene virus infections and develop antiviral treatments on the future avenue of antiviral research.

## Conclusion

Despite the indistinct mechanisms, RNA modifications currently are identified to affect the infection of diverse kinds of viruses, in which the antiviral innate immunity is the most prevalent factor. In the near future, some RNA modifications, including m^6^A and m^5^C, may serve as crucial targets for the rational design of improved live attenuated vaccine candidates. Importantly, considering the complex effects of epigenetic modifications in host cell transcriptome, developing these types of antiviral drugs or vaccines still needs additional studies to confirm such assumptions.

## Author Contributions

JT, WZ, and GQ: conceptualization and writing, review, and editing. JT and WZ: data curation and writing original draft. YC, QY, and N-NQ: visualization. All authors contributed to the article and approved the submitted version.

## Funding

This work was supported by Natural Science Foundation of Hebei Province, China (C2021201010), Hundreds Talents Program of Hebei Province, China (E2020050011), and Advanced Talents Incubation Program of the Hebei University (521000981413) to JT, the National Natural Science Foundation of China (31971227), the Science and Technology Project of Hebei Education Department (ZD2021010), and the Natural Science Foundation of Hebei Province, China (C2020201039).

## Conflict of Interest

The authors declare that the research was conducted in the absence of any commercial or financial relationships that could be construed as a potential conflict of interest.

## Publisher’s Note

All claims expressed in this article are solely those of the authors and do not necessarily represent those of their affiliated organizations, or those of the publisher, the editors and the reviewers. Any product that may be evaluated in this article, or claim that may be made by its manufacturer, is not guaranteed or endorsed by the publisher.

## Supplementary Material

The Supplementary Material for this article can be found online at: https://www.frontiersin.org/articles/10.3389/fmicb.2022.845625/full#supplementary-material
