# Supplementary material for: The Emerging Role of RNA Modifications in the Regulation of Antiviral Innate Immunity
Source: Front Microbiol. 2022 Feb 3;13:845625. doi: 10.3389/fmicb.2022.845625 (PMC8851159; doi:10.3389/fmicb.2022.845625)
Supplement: Supplementary file 1 [file Data_Sheet_1.PDF]

Table S1. List of RNA modifications in regulating virus infection

Modifications-virus	References
**m^6A**	
HIV-1	(Lichinchi, Gao et al. 2016; Kennedy, Bogerd et al. 2017; Tirumuru, Zhao et al. 2017; Lu, Tirumuru et al. 2018; Tirumuru and Wu 2019)
Avian sarcoma virus, Rous sarcoma virus	(Stoltzfus and Dane 1982; Kane and Beemon 1985)
Murine leukemia virus	(Courtney, Tsai et al. 2019; Courtney 2021)
Influenza virus	(Krug, Morgan et al. 1976; Narayan, Ayers et al. 1987; Courtney, Kennedy et al. 2017)
Flavivirus genus, Poliovirus, Enterovirus 71	(Gokhale, McIntyre et al. 2016; Lichinchi, Gao et al. 2016; McIntyre, Netzband et al. 2018; Hao, Hao et al. 2019)
Vesicular stomatitis virus, SARS-COV-2, Sendai virus, Mink enteritis parvovirus, HBV, HCV, encephalon myocarditis virus, herpes simplex virus type 1, Human metapneumovirus	In the present review.
**m^5C**	
Zika virus, Dengue virus, HCV, Poliovirus, HIV-1	(McIntyre, Netzband et al. 2018)
Murine leukemia virus	(Courtney, Tsai et al. 2019)
Sindbis virus, Drosophila C virus, Influenza virus	In the present review.
**2’-O-Me/m^7G**	
Viruses within *Corona-, Arteri-, Rhabdo-, Filo-, Paramyxo-, Pox-, and Reo-, Retro-, Togaviridae* families, flavivirus genus	(Decroly, Ferron et al. 2011; Decroly and Canard 2017)
Zika virus, Dengue virus, HCV, Poliovirus,HIV-1 Murine leukemia virus	(Dong, Chang et al. 2012; McIntyre, Netzband et al. 2018; Courtney, Tsai et al. 2019)
HIV-1, Viruses within *Corona-, Arteri-, Rhabdo-, Filo-, Paramyxo-, Pox-, Reoviridae* families and flavivirus genus	In the present review.
**A-to-I**	
HIV-1,HCV, Hepatitis δ virus	(Taylor, Puig et al. 2005; Casey 2006; Phuphuakrat, Kraiwong et al. 2008; Doria, Neri et al. 2009; McIntyre, Netzband et al. 2018)
Zika virus, Dengue virus, and Poliovirus	(Khrustalev, Khrustaleva et al. 2017; Piontkivska, Frederick et al. 2017; McIntyre, Netzband et al. 2018)
Influenza virus, Mink enteritis parvovirus, Vesicular stomatitis virus, Yellow fever virus, Chikungunya virus, Venezuelan equine encephalitis virus, Borna disease virus, Measles virus, Respiratory syncytial virus	In the present review.

References

Casey, J. L. (2006). "RNA editing in hepatitis delta virus." Curr Top Microbiol Immunol 307: 67-89.

Courtney, D. G. (2021). "Post-Transcriptional Regulation of Viral RNA through Epitranscriptional Modification." Cells 10(5).

Courtney, D. G., E. M. Kennedy, et al. (2017). "Epitranscriptomic Enhancement of Influenza A Virus Gene Expression and Replication." Cell Host Microbe 22(3): 377-386 e375.

Courtney, D. G., K. Tsai, et al. (2019). "Epitranscriptomic Addition of m(5)C to HIV-1 Transcripts Regulates Viral Gene Expression." Cell Host Microbe 26(2): 217-227 e216.

Decroly, E. and B. Canard (2017). "Biochemical principles and inhibitors to interfere with viral capping pathways." Curr Opin Virol 24: 87-96.

Decroly, E., F. Ferron, et al. (2011). "Conventional and unconventional mechanisms for capping viral mRNA." Nat Rev Microbiol 10(1): 51-65.

Dong, H., D. C. Chang, et al. (2012). "2'-O methylation of internal adenosine by flavivirus NS5 methyltransferase." PLoS Pathog 8(4): e1002642.

Doria, M., F. Neri, et al. (2009). "Editing of HIV-1 RNA by the double-stranded RNA deaminase ADAR1 stimulates viral infection." Nucleic Acids Res 37(17): 5848-5858.

Gokhale, N. S., A. B. R. McIntyre, et al. (2016). "N6-Methyladenosine in Flaviviridae Viral RNA Genomes Regulates Infection." Cell Host Microbe 20(5): 654-665.

Hao, H., S. Hao, et al. (2019). "N6-methyladenosine modification and METTL3 modulate enterovirus 71 replication." Nucleic Acids Res 47(1): 362-374.

Kane, S. E. and K. Beemon (1985). "Precise localization of m6A in Rous sarcoma virus RNA reveals clustering of methylation sites: implications for RNA processing." Mol Cell Biol 5(9): 2298-2306.

Kennedy, E. M., H. P. Bogerd, et al. (2017). "Posttranscriptional m(6)A Editing of HIV-1 mRNAs Enhances Viral Gene Expression." Cell Host Microbe 22(6): 830.

Khrustalev, V. V., T. A. Khrustaleva, et al. (2017). "Mutational Pressure in Zika Virus: Local ADAR-Editing Areas Associated with Pauses in Translation and Replication." Front Cell Infect Microbiol 7: 44.

Krug, R. M., M. A. Morgan, et al. (1976). "Influenza viral mRNA contains internal N6-methyladenosine and 5'-terminal 7-methylguanosine in cap structures." J Virol 20(1): 45-53.

Lichinchi, G., S. Gao, et al. (2016). "Dynamics of the human and viral m(6)A RNA methylomes during HIV-1 infection of T cells." Nat Microbiol 1: 16011.

Lu, W., N. Tirumuru, et al. (2018). "N(6)-Methyladenosine-binding proteins suppress HIV-1 infectivity and viral production." J Biol Chem 293(34): 12992-13005.

McIntyre, W., R. Netzband, et al. (2018). "Positive-sense RNA viruses reveal the complexity and dynamics of the cellular and viral epitranscriptomes during infection." Nucleic Acids Res 46(11): 5776-5791.

Narayan, P., D. F. Ayers, et al. (1987). "Unequal distribution of N6-methyladenosine in influenza virus mRNAs." Mol Cell Biol 7(4): 1572-1575.

Phuphuakrat, A., R. Kraiwong, et al. (2008). "Double-stranded RNA adenosine deaminases enhance expression of human immunodeficiency virus type 1 proteins." J Virol 82(21): 10864-10872.

Piontkivska, H., M. Frederick, et al. (2017). "RNA editing by the host ADAR system affects the molecular evolution of the Zika virus." Ecol Evol 7(12): 4475-4485.

Stoltzfus, C. M. and R. W. Dane (1982). "Accumulation of spliced avian retrovirus mRNA is inhibited in S-adenosylmethionine-depleted chicken embryo fibroblasts." J Virol 42(3): 918-931.

Taylor, D. R., M. Puig, et al. (2005). "New antiviral pathway that mediates hepatitis C virus replicon interferon sensitivity through ADAR1." J Virol 79(10): 6291-6298.

Tirumuru, N. and L. Wu (2019). "HIV-1 envelope proteins up-regulate N (6)-methyladenosine levels of cellular RNA independently of viral replication." J Biol Chem 294(9): 3249-3260.

Tirumuru, N., B. S. Zhao, et al. (2017). "Correction: N(6)-methyladenosine of HIV-1 RNA regulates viral infection and HIV-1 Gag protein expression." Elife 6.
